# Purulent Ophthalmia of Armies

**Published:** 1861-08

**Authors:** E. L. Holmes

**Affiliations:** Chicago


### CHICAGO
### MEDICAL JOURNAL.

VOL. IV.] AUGUST, 1861. [NO. 8.

### ORIGINAL COMMUNICATIONS.

### PURULENT OPHTHALMIA OF ARMIES.

BY E. L. HOLMES, M.D., of Chicago.

Comparatively few members of the profession in this country have acquired extensive experience in the practice of Military Surgery, and, probably, for this reason our medical writers have contributed little to enrich the literature of this branch of our science. Until very recently scarcely a single valuable work upon the subject had been produced by an American author.

Although the works recently published will undoubtedly be of great benefit to surgeons so suddenly called without previous experience in camp life, to watch over the health of our soldiers and treat the diseases and injuries peculiar to them, we apprehend neither the present nor the future wants of the military surgeon will be wholly supplied by these manuals. They have evidently been hastily prepared for the sudden emergencies in which the surgeons of our volunteer regiments are placed, and are undoubtedly, under the circumstances, as comprehensive as could have been expected. The great increase in the number of soldiers, however, which the necessities of the country may for some time require, will but too soon, we fear, furnish an extensive field of observation and practice, which will enable our military surgeons to supply, if they choose, this deficiency in American medical literature.

Among the subjects which have been neglected in the treatises already published, we would mention "Ophthalmie Militarii" or "Purulent Ophthalmia of Armies." And yet this is one of the most important matters to which the attention of the military surgeon can be directed. Any one who is acquainted with certain facts in the history of military hygiene in the European armies during the past seventy years, must be convinced of the truth of this statement. For these facts we would refer to the various standard works on Diseases of the Eye, especially that of Lawrence, and several excellent monographs published on the continent of Europe.

It is reported officially that in the year 1830, one-eighth of the Belgian army was disabled with this disease, and in 1840 there were 5000 cases in the army consisting of 50,000 men. More than *one hundred thousand* cases had been reported in the Belgian army since the first appearance of the disease. In some regiments more than one-half of the soldiers were affected.

Within the space of eight years 30,000 cases occurred in the Prussian army.

In the year 1812, 1500 cases occurred in the garrison of Ancona.

From the reports of two English hospitals, it appears that in Dec., 1810, 2317 soldiers were a burden to the public from blindness produced by ophthalmia, and that a large number in addition had lost one eye.

Dr. Vetch informs us that in the 2d battalion of the 52d, numbering about 700 men, 636 cases of purulent ophthalmia occurred in one year.

Assalini states that two-thirds of the French army were affected at one time.

Official reports abound in accounts of the ravages of this terrible disease in hospitals, in fortresses, on board ship and in schools; but we need not multiply examples.

Well might the subject have deeply interested the European governments when so large a proportion of their soldiers were disabled, and, in too many instances, thrown upon public charity, blind and dependant for life. In view of the disastrous effects of this disease, several governments offered prizes for the best essays upon the subject, and appointed at different times boards of commissioners, composed of the most experienced Ophthalmologists, to investigate its causes, treatment and prevention.

We see little reason for supposing our own armies will not suffer to a greater or less extent from this evil. There will probably be for months, if not for years, not less than 400,000 troops in the armies of the Federal Government. From exposure, improper food and insufficient dress, evils incident to military operations, these men will be peculiarly liable to nearly every form of disease.

As is well known by experienced physicians, who have practised in the South and West, Ophthalmia is often epidemic in certain localities, in which portions of our army will be in active service. It should be remembered by the military surgeon, that whenever even a single soldier in a regiment is affected with this disease, it is almost inevitably communicated by contagion to his comrades. This takes place not only by direct contact through carelessness in the use of towels and general neglect as regards habits of cleanliness, but also, as some writers believe, by the influence of the atmosphere of tents and barracks impregnated with particles of purulent matter.

We may seem to our readers inclined to seek danger and trouble where none exist. The remarkable facts, however, in the history of European armies, above given, certainly show that there are good grounds for fear. An intelligent surgeon of this State has lately informed us that several cases of severe Ophthalmia have already appeared in the regiment under his charge.

Upon the care and skill of our surgeons in treating Purulent Ophthalmia, will depend, we believe, the services of a large number of our soldiers. Upon this same care and skill will depend whether those soldiers affected with this disease shall be restored to health or returned home blind and dependent upon friends or the public for support. As a matter of public economy, to say nothing of humanity, every effort should be made to preserve the health and thereby the efficiency of the army, but also to reduce the present expense of maintaining a large number of invalids and the future expense of supporting the blind; the Government should therefore endeavor to appoint such surgeons only as are in all respects competent to fill so important positions.

It is not our present purpose to attempt to supply any deficiency on the particular point in question which may occur in our works on Military Surgery. We propose at some future time to offer the readers of the JOURNAL, if desired, a paper on Conjunctivitis as it prevails in the Northwest. We have been much interested for the past few years in collecting facts relative to this troublesome disease, which we trust will not prove wholly uninteresting to the profession at large.

We wish, however, to offer a single suggestion, which we consider of much practical value in reference to the mode of applying strong caustic solutions to the eye in the early stages of Purulent or Catarahal Ophthlmia. We are fully convinced that many physicians often fail to cut short these diseases by applying solutions of insufficient strength and also do direct injury by improper modes of applying the stronger solutions. In a large proportion of uncomplicated cases, when the inflammation is confined principally to the palpebral conjunctiva, rendering them highly vascular and œdematous, we consider the use of solutions of argenti nitras, varying in strength from ℈j ℈ij or even ℈iiss to the ounce of water, as most reliable, *provided they are properly applied.* We never drop such collyria under the lids, for, thus introduced, they are to a certain extent neutralized by the salts of the secretions and prevented from passing equally over the whole surface of the conjunctiva by the spasmodic contraction of the lids.

To the conjunctiva of both lids exposed as much as possible by inverting the upper lid and drawing down the lower lid, we first press gently a piece of dry linen for the purpose of removing all moisture and mucous. Upon the whole surface thus prepared we paint some one of the solutions above mentioned by means of a large but short camel's hair, *thoroughly moistened* in the solution and yet *not saturated* with it. The brush should be passed freely over the surface till the whole conjunctiva presents the characteristic white appearance produced by Argenti Nitras, after which the moisture should be again removed with the linen and the lids returned to their natural position. One application of this kind a day is usually sufficient. The stronger the solution, the less freely it should be applied to the inflamed surface.

We have followed this method with such good results in so many cases that we would recommend it to all who have never tried it, where strong caustic solutions are indicated.

In conclusion, we would again urge upon our Military Surgeons the careful consideration of this subject. We would remind them of the necessity of prompt treatment in the very earliest stages of the disease, and of immediate isolation of affected soldiers.

A new field of observation is undoubtedly open to members of our profession, and whoever will collect the most reliable facts regarding the causes, progress and treatment of Ophthalmia as found in our armies, will perform an important work for the profession and secure for himself an enviable reputation.

